# Landscape Level Variation in Tick Abundance Relative to Seasonal Migration in Red Deer

**DOI:** 10.1371/journal.pone.0071299

**Published:** 2013-08-09

**Authors:** Lars Qviller, Nina Risnes-Olsen, Kim Magnus Bærum, Erling L. Meisingset, Leif Egil Loe, Bjørnar Ytrehus, Hildegunn Viljugrein, Atle Mysterud

**Affiliations:** 1 Centre for Ecological and Evolutionary Synthesis, Department of Biosciences, University of Oslo, Oslo, Norway; 2 Norwegian Institute for Agricultural and Environmental Research, Organic food and farming Division, Tingvoll, Norway; 3 Norwegian University of Life Science, Department of Ecology and Natural Resource Management, Aas, Norway; 4 Norwegian Veterinary Institute, Oslo, Norway; Université de Sherbrooke, Canada

## Abstract

Partial migration is common among northern ungulates, typically involving an altitudinal movement for seasonally migratory individuals. The main driving force behind migration is the benefit of an extended period of access to newly emerged, high quality forage along the green up gradient with increasing altitude; termed the forage maturation hypothesis. Any other limiting factor spatially correlated with this gradient may provide extra benefits or costs to migration, without necessarily being the cause of it. A common ectoparasite on cervids in Europe is the sheep tick (*Ixodes ricinus*), but it has not been tested whether migration may lead to the spatial separation from these parasites and thus potentially provide an additional benefit to migration. Further, if there is questing of ticks in winter ranges in May before spring migration, deer migration may also play a role for the distribution of ticks. We quantified the abundance of questing sheep tick within winter and summer home ranges of migratory (n = 42) and resident red deer (*Cervus elaphus*) individuals (n = 32) in two populations in May and August 2009–2012. Consistent with predictions, there was markedly lower abundance of questing ticks in the summer areas of migrating red deer (0.6/20 m^2^), both when compared to the annual home range of resident deer (4.9/20 m^2^) and the winter home ranges of migrants (5.8/20 m^2^). The reduced abundances within summer home ranges of migrants were explained by lower abundance of ticks with increasing altitude and distance from the coast. The lower abundance of ticks in summer home ranges of migratory deer does not imply that ticks are the main driver of migration (being most likely the benefits expected from forage maturation), but it suggests that ticks may add to the value of migration in some ecosystems and that it may act to spread ticks long distances in the landscape.

## Introduction

Migration is a well-known feature of animals in areas with strong seasonal shifting in limiting factors [1]. For large herbivores, a main driver of migration is spatiotemporal variation in food resources [2]. In the tropics, large herbivore migrations often follow rainfall cycles triggering migration between grasslands in the wet seasons and forests in the dry seasons [2]. Migration patterns of northern ungulates are often more subtle, and most populations are partly migratory [3]–[5]. Many red deer in Norway move from coast to inland and from low to high elevation, while other individuals remain resident [6]. Migrating individuals of red deer benefit in terms of plant quality as predicted by the forage maturation hypothesis [7], [8]. Although red deer migration follows predictions from the forage maturation hypothesis, they have been found to migrate faster between the seasonal ranges than predicted from the plant phenological development [8]. One possible explanation is that altitudinal migration of northern cervids is affected by other mechanisms than plant phenology [6].

It is well established that many deer species migrate to areas with lower risk of predation [1], [9]. In contrast, the hypothesis that seasonal variation in the spatial distribution of parasites may play a role for migration of northern ungulates has less well tested [10], [11]. As a result of this, the possible beneficial aspects of red deer migration with respect to parasites lack empirical evidence. Because of the harmful nature of parasites, host species tend to develop not only physiological but also behavioural defences like avoiding areas with high parasite densities [12]. Large herbivores may evade endoparasites by keeping off areas with abundant faeces droppings [13], [14]. Harassment from ectoparasites play a role for activity of reindeer (*Rangifer tarandus*) [15], but insect relief could not explain spring migration of caribou [16]. Cattle (*Bos taurus*) refused to graze in paddocks seeded with large numbers of tick larvae [17], and experiments show that cattle get lower tick burden than expected from a direct proportional relationship with tick densities in the paddocks, indicating some small scale avoidance of ticks [18]. Roe deer may be infested by several hundred ticks [19]. Due to the high infestation levels of ticks on deer, a further examination of the relationship of tick and deer is required.

In Europe, one of the most common ectoparasites of red deer (*Cervus elaphus*) is the sheep tick (*Ixodes ricinus*). This three stage tick spends most of its life as a free-living surface dwelling parasite, making it strongly dependent on climatic conditions [20]. It is dependent on a blood meal for further development from larva to nymph, from nymph to adult and finally, the adult females need a large blood meal to manage to produce about 2000 eggs. The tick quest in the vegetation between stages until a host passes by, then cling to the host, find a suitable place for sucking, and engorge for a period of a few days up to two weeks [21]. The distribution and abundance of the tick *Ixodes ricinus* has increased both on the west coast of Norway and in Scandinavia in general [22]–[24]. The relationship between tick abundance and red deer migration is interesting from two perspectives: If migration leads to spatial separation from ticks, it may add to the benefits of migration by lowering risk of parasitism. Further, migratory red deer may act as a vehicle to move ticks around in the landscape, if there are questing ticks around timing of migration in spring, since the duration of migration is often shorter (frequently 1–2 days; mean 5 days [6]) than the attachment period.

Based on data available from 74 GPS-marked red deer, we have surveyed the vegetation in winter and summer home ranges of resident and migratory red deer for ticks with the cloth lure method over 4 years (2009–2012). As deer migration is often from coast to inland and from low to high elevation, we tested the hypothesis that tick abundances would be lower in summer home ranges of migratory deer as compared to winter ranges and home ranges of resident red deer. In addition, for migratory red deer to function as a vehicle for ticks, the ticks must have started some activity prior to migration in order to move ticks from high to low tick density areas.

## Materials and Methods

### Ethics Statement

This study involves using existing information from GPS-marked red deer individuals from ongoing studies [6], [8]. Individuals were marked with standard GPS-collars from either Televilt/Followit (Stockholm, Sweden) or Vectronic (Berlin, Germany). All marking procedures have been approved by the Norwegian Animal Research Authority (termed “Forsøksdyrutvalget” in Norwegian) and by the Directorate for Nature Management for all locations. For each location, the specific private landowner gave his permission to mark animals in all cases. Our study thus adheres to the “Guidelines for the Use of Animals in Research”, and to the legal requirements of Norway where the work has been carried out. The field studies did not involve endangered or protected species.

### Study Areas

**Figure 1:**
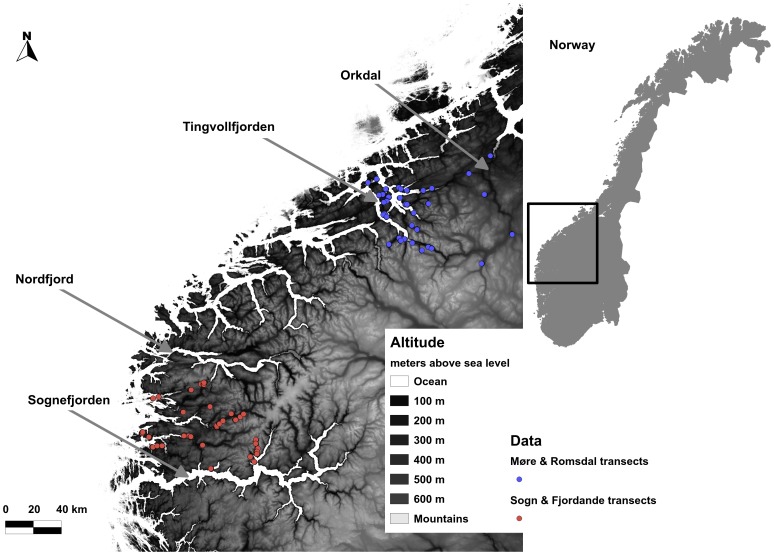
A map over the study area along the west coast of Norway showing the distribution of transects. The blue dots represents transects from the Møre & Romsdal data set, and the red dots represents transects from the Sogn & Fjordane data set. Lighter shades of grey represent increasing altitude up to 2469 m a.s.l.

Data was collected in two distinct study areas along the west coast of Norway. The first is in Sogn & Fjordane county limited by Sognefjorden in the south and Nordfjord in the north (Fig. 1). The climate is characterized by cool summers and mild winters. This area had an average yearly precipitation of 2270 mm and an average temperature of 6.0°C between 1961 and 1990 (http://met.no; Norwegian meteorological station no. 57170). The second area is situated in the northern parts of Møre & Romsdal county, crossing over the border to Sør-Trøndelag county. It is limited by Tingvollfjorden in the west and Orkdal in the east (Fig. 1). This area had an average annual temperature of 5.6°C and 1160 mm annual precipitation (http://met.no; Norwegian meteorological station no. 64550).

The vegetation in the study area lies within the boreonemoral vegetation zone [25]. Forests are dominated by Scots pine (*Pinus sylvestris*), alder (*Alnus incana*) and birch (*Betula* spp.), with scattered stands of Norway spruce *(Picea abies*) from extensive planting by forestry [26]. The topography is rugged, with plateaus and summits above 1000 m a.s.l. few kilometers from the sea. Climate is generally colder with increasing altitude and distance from the coast. The red deer population has increased markedly over the last decades along the south west coast of Norway [27]. In the hunting season 2010/2011 (10^th^ of Sept.–15^th^ of Nov.), a total of 11771 and 11138 red deer was harvested in Sogn & Fjordane and Møre & Romsdal, respectively (Statistics Norway 2012).

Roe deer (*Capreolus capreolus*) and moose (*Alces alces*) are present in Møre & Romsdal, but absent in the study area in Sogn & Fjordane. There is seasonal grazing by livestock in several parts of the study areas [28].

### Sampling Design

Our study was facilitated by prior knowledge of seasonal home ranges of 17 resident and 24 migratory female red deer in Sogn & Fjordane, and 13 resident (12 females and 1 males) and 19 migratory (10 males and 9 females) red deer in Møre & Romsdal, Norway. We placed transects for collecting tick abundance crossing the middle of each seasonal home range (95% Kernels). For resident animals, by definition having overlapping seasonal home ranges, there was one transect, while there were two transects for migratory animals; one in the winter home range and one in the summer home range.

Several of the deer had common winter ranges (assessed by overlapping home ranges) being represented by one transect (32 winter/resident home ranges on 19 transects in Møre & Romsdal, 41 winter/resident home ranges on 12 transects in Sogn & Fjordane. Due to the presence of both resident and migratory deer within the same winter range, we chose to include all deer from a given winter range as they had non-overlapping summer ranges and indicate a strong degree of individual choice. To ensure that our results are not driven by this repeated use of data from a common few winter ranges, we used a bootstrap procedure explained under statistical methods.

We sampled a total of 34 home range areas for tick abundance in Sogn & Fjordane and 42 in Møre & Romsdal. Transects were laid along the main gradient in altitude and crossing the center of each home range. We placed 12 survey plots along each transect with randomized distances between 20 and 50 m. The main data were collected in May, which is the main questing period of ticks (unpubl. data) and around onset of the main migration period of red deer. In addition, we also collected similar data in August to ensure that later tick phenology at high altitudes are not driving the patterns observed. We sampled the same transects in 2009, 2010 (only May), 2011 and 2012 in the Sogn & Fjordane study area, while sampling was performed 2011 and 2012 in the Møre & Romsdal study area. Note that data from GPS-marked animals were collected from years prior to the sampling, but the large majority of adult red deer are known to follow the same migration pattern every year (Meisingset, Loe and Mysterud, unpubl. data). We therefore also tested whether the pattern of tick distribution was stable over years. Exact UTM coordinates were registered using a handheld Garmin GPSmap 60CSx at every visit to the sampling station.

### Sampling Procedure – the Cloth Lure Method

The abundance of *I. ricinus* ticks were sampled from the vegetation using the cloth lure method [29]. A towel was attached to the end of a rod, as a flag, and the towel was dragged over the vegetation, allowing the questing ticks to attach (termed “flagging”). Each of the 12 survey plots covered an approximately 10 m long and a 2 m wide belt; i.e. 20 m^2^. We used 50*100 cm towels, which were frequently replaced with new ones if they became wet. Ticks were counted and removed from the towel for every 2^nd^ m after two drags on each side of the towel. Number of adult females, adult males and nymphs were registered for each survey plot. Some survey plots at higher altitude were still covered in snow some years and counted as zero. The cloth lure method typically underestimates the true abundance in an area [30]. However, our aim is to compare relative abundance within home range for which the method is suitable.

### Geographical Covariates

All terrain data were retrieved from a 100×100 m scaled topographic map with the GRASS GIS-software [31]. We retrieved altitude, slope, and two different measures of distance to the ocean. The Norwegian coast is broken by many long fjords, and the innermost part of Sognefjorden is relatively sheltered from the open ocean. We have therefore quantified both the distance to the fjord, which is the Euclidean distance to the closest contact with sea water, and distance to the coast, which is the Euclidean distance to the outer Norwegian coastline.

### Statistical Analyses

All statistical analyses were done with the R statistical software version 3.0.1 [32]. First, we built a model of tick abundance relative to terrain properties (altitude, slope and two measures of distance from the coast as fixed effects) to get an understanding of tick distribution and why home range types might differ in tick abundance. Only May data were used in this analysis due to larger sample size. Second, we built a model to test whether there were differences in tick abundance between home range types (home range of resident individuals, winter and summer range of migratory individuals). This model included home range category, sex, year and their interactions as fixed effects. Both models used number of ticks as the response variable. The two study areas were analyzed separately, because the time series are of unequal length. Since data from August were missing in Sogn & Fjordane for 2010, we also present in main results analyses of total counts from May and August separately and with nymphs and adults pooled. Additional analyses with separation of life stages are presented in the supporting information, but yield the same overall pattern.

There were three main challenges related to the analyses of the tick abundance data. Parasite abundance data are often overdispersed relative to what is expected from a Poisson distribution, therefore a negative binomial distribution is often used [33], [34]. In addition, it is fairly frequent with a higher proportion of zeros than expected even from a negative binomial distribution [34], warranting the use of zero-inflated models [35]. Initial analyses of our data confirmed that models including a zero-inflated negative binomial distribution gave the best fit.

The second challenge relates to the sampling design yielding a nested data structure, requiring use of mixed models. We used the library glmmADMB that is able to tackle zero-inflated negative binomial distribution within a mixed model setting [36].

The third challenge was that some red deer had overlapping winter home ranges. We employed a bootstrap procedure, where individual deer from common winter range (i.e. the same transect) were removed at random before the analysis was performed. We then replicated the procedure 100 times for each model. Due to computational challenges, only the final models were subject to bootstrapping.

We used different random terms for the home range model and the terrain model. For the home range model, we used the individual red deer as a random term to handle the nested structure of the data. For the terrain model, we used transect as a random term. The zero inflation option was used in both home range and terrain models, i.e., the zeros are modeled as coming from two different processes: the binomial and the count process.

We explored non-linear relationships and the need for polynomial terms in subsequent analyses with generalized additive models, using the mgcv-library [37]. Model selection was done with the Akaike Information Criterion [38].

## Results

A total of 8438 ticks were found in Sogn & Fjordane (2161 in 2009, 2092 in 2010, 1498 in 2011 and 1803 in 2012) and 5354 ticks in Møre & Romsdal (2371 in 2011 and 2983 in 2012) in May.

In August, a total of 2946 ticks were found in Sogn & Fjordane (1319 in 2009, 667 in 2011 and 960 in 2012) and 3856 ticks in Møre & Romsdal (1917 in 2011 and 1939 in 2012).

### Terrain Model

**Figure 2:**
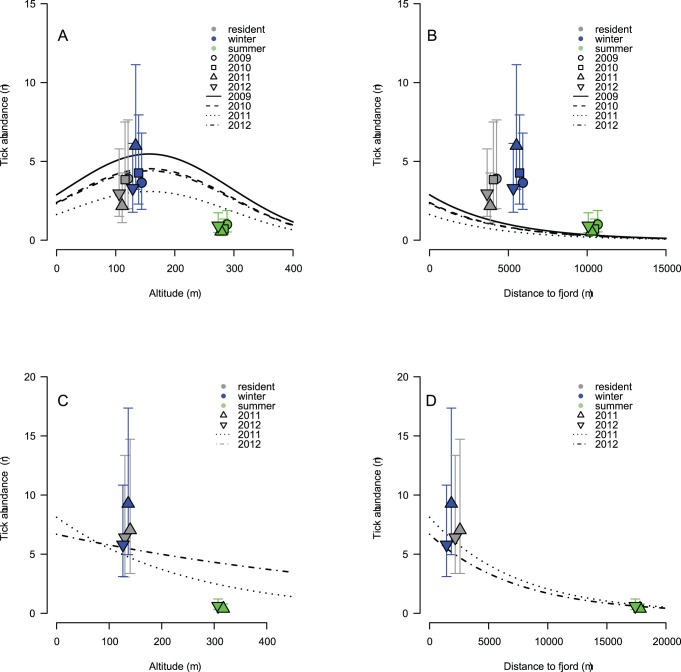
Tick abundance as a function of (A, C) altitude in meters above sea level and (B, D) distance to fjord measured in meters in (A, B) Sogn & Fjordane and (C, D) Møre & Romsdal counties, Norway. The symbols are estimates of the three home range categories (±1.96*SE) plotted against mean altitude and distance to fjord within the category. Gray symbols represent home ranges of resident red deer, blue and green symbols represents winter and summer home ranges, respectively, for migratory red deer. The home ranges are identical between years, but we have shifted the home range symbols slightly between years for visibility in the figure. Note that lines and point estimates come from different models and therefore do not match entirely due to other factors included (altitude, inclination and distance to fjord).

Tick counts from the terrain in May was best predicted by a model including year, altitude, (altitude)^2^, distance to coast and slope as predictors in Sogn & Fjordane (Table 1). The tick abundance decreased with increasing altitude. The inclusion of a 2^nd^ order term for altitude suggested a weak non-linear relationship (Fig. 2A). Tick abundance was quite high at low altitude, but with a slight peak at an altitude of 156 m, and a marked decline thereafter. In Møre & Romsdal, the tick abundance was best explained by year, slope, distance to fjord, altitude and the year:altitude interaction. The tick abundance was decreasing with increasing altitude with different slopes between the two years (Table 2 and 3, Fig. 2C). The highest elevation with recorded tick presence in both areas was at 545 m a.s.l. Increased distance to fjord and decreased inclination were linked to a lower abundance of ticks in both areas (Table 3, Fig. 2B and 2D). The year interactions with distance to fjord and inclination did not enter the most parsimonious models in any of the two areas (Table 1 and 2). Though the interaction between year and altitude was significant for Møre & Romsdal (Table 3), it did not alter the pattern of decreasing tick abundance with increasing altitude (Fig. 2C). Thus, the main spatial distribution pattern of questing ticks remained similar between years.

**Table 1:** Model selection for the model explaining variation in abundance of ticks as a function of landscape characteristics in Sogn & Fjordane, Norway, years 2009–2012.

Altitude	(Altitude)^2^	Distance to fjord	Log(distance)	Slope	Distanceto coast	Year	Altitude:year	(Altitude)^2^:year	AIC	ΔAIC
x									6068.9	103.8
		x							6043.3	78.2
			x						6069.7	104.6
				x					6045.4	80.3
						x			6048.7	83.6
					x				6066.0	100.9
x		x							6045.0	79.9
		x				x			6024.1	59.0
		x	x						6044.3	79.2
		x		x					6015.3	50.2
		x			x				6045.2	80.1
x		x		x					6009.1	44.0
		x		x		x			5996.3	31.2
		x		x	x				6016.8	51.7
x		x		x		x			5991.6	26.5
		x		x	x	x			5997.8	37.2
x		x		x	x	x			5993.0	27.9
x	x	x		x	x	x			5966.6	1.5
**x**	**x**	**x**		**x**		**x**			**5965.1**	**0**
x	x	x		x		x	x		5968.2	3.1
x	x	x		x		x		x	5968.6	3.5
x	x	x		x		x	x	x	5972.9	7.8
x	x	x		x	x	x	x	x	5974.4	9.3

The best model (ΔAIC = 0) is presented in bold fonts. x = term included in model.

**Table 2:** Model selection for the model explaining variation in abundance of ticks as a function of landscape characteristics in Møre & Romsdal for 2011 and 2012.

Altitude	(Altitude)^2^	Distance to fjord	log(distance)	Slope	Distance to coast	Year	Altitude:Year	AIC	ΔAIC
x								3944.0	43.4
		x						3910.2	9.6
			x					3945.5	44.9
				x				3954.6	54.0
					x			3927.8	27.2
						x		3951.7	51.1
x		x						3909.4	8.8
		x				x		3908.4	7.8
		x						3910.2	9.6
		x		x				3910.9	10.3
		x			x			3909.8	9.2
x		x				x		3907.6	7.0
		x		x		x		3908.9	8.3
		x			x	x		3908.1	7.5
x		x		x		x		3905.7	5.1
		x			x	x		3908.2	7.6
x	x	x				x		3909.0	8.4
x		x		x	x	x		3905.8	5.2
x	x	x		x		x		3906.9	6.3
x	x	x		x	x	x		3907.2	6.6
**x**		**x**		**x**		**x**	**x**	**3900.6**	**0**
x	x	x		x		x	x	3901.8	1.2
x	x	x		x	x	x	x	3902.1	1.5
x		x		x	x	x	x	3900.7	0.1

The best model (ΔAIC = 0) is presented in bold fonts. x = term included in model.

**Table 3:** Estimates from the best model explaining variation in abundance of ticks as a function of landscape characteristics in Sogn & Fjordane and Møre & Romsdal counties, Norway.

Parameter	Estimate	S.E.	z	P
Sogn & Fjordane				
Intercept	1.1	0.33	3.2	<0.001
Year 2010 vs. 2009	−0.2	0.11	−1.7	0.091
Year 2011 vs. 2009	−0.6	0.12	−4.9	<0.001
Year 2012 vs. 2009	−0.2	0.11	−1.9	0.054
Slope	0.031	6.2e-03	5.1	<0.001
Distance to fjord	−2.1e-04	3.3e-05	−6.4	<0.001
Altitude	8e-03	2.2e-03	3.8	<0.001
(Altitude)^2^	−2.6e-05	5.0e-06	−5.2	<0.001
Møre & Romsdal				
Intercept	2.1	0.29	7.2	<0.001
Year 2012 vs. 2011	−0.2	0.19	−1.0	0.300
Slope	0.019	9.2e-03	2.1	0.039
Distance to fjord	−1.4e-04	2.5e-05	−5.6	<0.001
Altitude	−4e-03	1.2e-03	−3.2	<0.001
Altitude:Year	−2.5e-03	9.3e-04	2.6	<0.001

Year is a factor variable. Baseline for year is 2009 in Sogn & Fjordane and 2011 in Møre & Romsdal. The models included transect as a random term.

### Home Range Model

The most parsimonious home range models included home range type, year (categorical) and the interaction between year and home range type as fixed variables for both study areas (Table 4). Summer home ranges of migratory red deer had significantly lower tick abundance than both winter home ranges and home ranges of resident animals for both study areas (Table 5, Fig. 2). Both male and female red deer were represented in Møre & Romsdal, but sex (or its interaction with the other terms) did not enter the best models. The estimated tick abundance varied annually, but there was consistently lower tick abundance in summer ranges of migratory red deer than in the two other categories across years (Fig. 2). Summer home ranges of migratory red deer had higher mean altitude (Fig. 2A and C), and were farther from the fjords than the winter and resident home ranges (Fig. 2B and D). The results were consistent when we ran analyses separately for nymphs and adults, and the main pattern of lower abundance of ticks in summer ranges were also present in August (Table 6 and 7, in the supporting information). The bootstrap procedure removing individuals randomly from transects with more than one deer, were qualitatively consistent and quantitative estimates were largely similar (Table 5 and 7, and Table S1 and S2).

**Table 4:** Results from model selection performed on tick abundance from the Sogn & Fjordane and Møre & Romsdal counties in Norway in May at the scale of red deer home ranges.

HRtype	Year	Sex	HRtype :Year	HRtype:Sex	Sex:Year	Hrtype:Sex:Year	AIC	ΔAIC
Sogn & Fjordane, *May*								
							14455.0	285.3
x							14217.4	47.6
	x						14457.7	288
x	x						14213.6	43.9
**x**	**x**		**x**				**14169.7**	**0**
Møre & Romsdal								
							5900.9	45.3
x							5869.9	14.3
	x						5897.2	41.6
		x					5902.5	46.9
	x						5870.4	14.8
x		x					5873.5	17.9
	x	x					5898.7	43.1
x	x	x					5872.4	16.8
**x**	**x**		**x**				**5855.6**	**0**
x		x		x			5874.0	18.4
	x	x			x		5894.5	38.9
x	x	x	x				5857.6	2.0
x	x	x		x			5874.3	18.7
x	x	x			x		5868.3	12.7
x	x	x	x	x			5859.6	4.0
x	x	x	x		x		5855.8	0.2
x	x	x		x	x		5870.2	14.6
x	x	x	x	x	x		5857.8	2.2
x	x	x	x	x	x	x	5861.4	5.8

HRtype = home range type (3 levels), year (4 or 2 levels). The models also included red deer ID as a random term.

**Table 5:** Estimates from the home range model from the Sogn & Fjordane and Møre & Romsdal, with the best AIC fit predicting tick abundance within home ranges of resident animals, and winter and summer home range of migratory red deer.

Parameter	Estimate	S.E.	z	p
*Sogn & Fjordane*				
Intercept	−4e-3	0.33	−0.0	0.990
HR (resident vs. summer)	1.4	0.40	3.5	<0.001
HR (winter vs. summer)	1.3	0.17	7.8	<0.001
Year 2010 vs. 2009	−0.3	0.15	−2.2	0.031
Year 2011 vs. 2009	−0.6	0.16	−3.6	<0.001
Year 2012 vs. 2009	0.07	0.16	−0.5	0.630
HR (resident vs. summer): Year 2010 vs. 2009	0.3	0.19	1.6	0.100
HR (resident vs. summer): Year 2011 vs. 2009	4e-3	0.20	−0.0	0.980
HR (resident vs. summer): Year 2012 vs. 2009	−0.2	0.20	−1.0	0.300
HR (winter vs. summer): Year 2010 vs. 2009	0.5	0.21	2.3	0.020
HR (winter vs. summer): Year 2011 vs. 2009	1.1	0.22	5.0	<0.001
HR (winter vs. summer): Year 2012 vs. 2009	0.02	0.21	−0.1	0.910
*Møre & Romsdal*				
Intercept	−0.9	0.36	−2.6	0.009
HR (resident vs. summer)	2.9	0.52	5.6	<0.001
HR (winter vs. summer)	3.2	0.48	6.6	<0.001
Year 2012 vs. 2011	0.4	0.17	2.6	0.011
HR (resident vs summer):Year	−0.5	0.23	−2.3	0.022
HR (winter vs summer):Year	−0.9	0.21	−4.4	<0.001

Note that all variables are factors. Baselines are “summer” home range of migratory animals and year 2009 in Sogn & Fjordane and year 2011 in Møre & Romsdal. Individual ID of each red deer was fitted as a random term. HR = home range type. All ticks stages are pooled in these analyses.

**Table 6:** Results from model selection performed on tick abundance from the Sogn & Fjordane and Møre & Romsdal counties in Norway in August at the scale of red deer home ranges.

HRtype	Year	HRtype :Year	AIC	ΔAIC
Sogn & Fjordane, *August*				
			8908.0	75.8
x			8855.1	22.9
	x		8886.2	54
x	x		8833.3	1.1
**x**	**x**	**x**	**8832.2**	**0**
				
Møre & Romsdal, *August*				
			5533.9	271.9
x			5271.4	9.4
	x		5527.1	265.1
x	x		5270.2	8.2
**x**	**x**	**x**	**5262.0**	**0**

HRtype = home range type (3 levels), year (4 or 2 levels). The models also included red deer ID as a random term.

**Table 7:** Parameter estimates and test statistics for all models of tick abundance relative to red deer home ranges.

Sogn & Fjordane, *August*	Estimate	S.E.	z	p
Intercept	**−0.2**	0.28	−0.8	0.412
HR (resident vs. summer)	**1.1**	0.36	3.0	0.003
HR (winter vs. summer)	**0.67**	0.091	7.4	<0.001
Year 2011 vs. 2009	−0.36	0.072	−5.0	<0.001
Year 2012 vs. 2009	−0.24	0.071	−3.3	<0.001
**Møre & Romsdal, ** ***August***	**Estimate**	**S.E.**	**z**	**p**
Intercept	**−0.3**	0.27	−1.3	0.192
HR (resident vs. summer)	**1.9**	0.41	4.5	<0.001
HR (winter vs. summer)	**2.0**	0.15	13.9	<0.001
Year 2012 vs. 2011	0.2	0.16	1.3	0.179
HR (resident vs summer):Year	−0.2	0.22	−0.9	0.355
HR (winter vs summer):Year	−0.6	0.20	−3.3	<0.001

Baselines are “summer” home range of migratory animals and year 2009 in Sogn & Fjordane and year 2011 in Møre & Romsdal. Individual ID of each red deer was fitted as a random term. HR = home range type. All ticks stages are pooled in these analyses.

## Discussion

Most northern ungulate populations are partially migratory [39]. It is well known that some individuals move from low elevation winter ranges to high elevation summer ranges [6], [8]. The autumn migration pattern is mainly due to snow accumulation at high elevation during winter [40], while the uphill spring migration is at least partly due to herbivores following the green-up altitude gradient in food quality, called the forage maturation hypothesis [2], [5], [7]. We found evidence for a lower abundance of *Ixodes ricinus* in the summer ranges of migratory red deer compared both to their winter ranges and the year around ranges of resident red deer. This indicates that migratory red deer also may benefit in terms of reduced parasite pressure from ticks. Further, since mean time of migration in spring coincide with the main questing period of ticks in May, migratory deer may also play a role for moving ticks over long distances each spring.

### Tick Distribution in the Landscape

Migration routes typically follow an altitudinal and/or coast-inland gradient [8], and the summer home ranges are localized farther inland and at higher altitudes than the winter ranges and the year round ranges of resident deer [6, this study]. In summer ranges of migratory red deer, we see a pattern of lower tick abundance that is partly an effect of increased altitude, distance to the coast and the inclination. These factors are climate proxies in some respect. It is widely accepted that an oceanic climate is favorable to ixodid ticks because of their temperature and humidity requirements [41]–[43]. Reduced temperature with increased altitude leads to shorter growth season and developmental constraints, while drought and changes in humidity saturation deficit increase mortality [44]–[47]. We found a decrease in tick abundance with increasing altitude and with increasing distance to the sea.

Altitude and distance to fjord in our terrain model follow the oceanic/continental gradient. The slightly lower abundance of ticks at the very lowest elevations in one study area (Sogn & Fjordane) are likely because some lowland shore areas are dry windblown meadows or areas with sparse forest cover in which ticks are mostly absent. Gilbert [47] reported reduced tick abundance as an effect of lower red deer density in addition to climatic factors along the altitudinal gradient. A similar result was found in a study on tick loads on roe deer (*Capreolus capreolus*) in Italy [48], and also small mammals seem to be an important factor for tick abundances [49]. The impact of red deer density on tick abundance has been made clear in a fencing experiment, where areas that excluded red deer had reduced numbers of *I. ricinus* [50]. Migration typically starts after the onset of tick activity. Ticks in winter/resident home ranges does therefore have access to much higher red deer density in the beginning of the questing period than the ticks in summer home ranges. This suggests two main mechanisms behind lower tick abundances in the summer home ranges of migratory deer: climatic constraints with increasing altitude and reduced cervid host availability. It is currently not known if also other host species (e.g. rodents), might be less abundant in summer home ranges due to the same climatic constraints.

Red deer may in some cases serve as vehicle for ticks [51]. Since ticks were questing in the winter ranges of migratory red deer prior to spring migration, our study confirm a potential role of deer as vehicles for ticks in our ecosystem. Migratory red deer have summer ranges in some areas representing likely the climatic border for ticks. Duration of migration [8] tend to be shorter than attachment period [21], and ticks that attach in winter areas will likely in many cases not detach until the red deer is in its summer home range. The annual spread of ticks by red deer during seasonal migration in spring may therefore lead to very rapid establishment in new areas with climate change.

### Are Ticks Harmful to Deer?

The lower abundance of ticks in home ranges of migratory deer does not imply that ticks are important for migration, since it also coincides with benefits from forage maturation. Nearly all red deer migrates even in eastern and south-eastern Norway [6], which are areas with low abundances of ticks or no ticks present at all [24], [41], [52]. Reduced tick abundance is therefore not likely the primary driver of migration, but ticks may nevertheless add to the benefit of migration. The most obvious direct cost of ticks is the blood lost during infestation. Some studies find significant losses in smaller hosts. Talleklint and Jaenson [53] report that roe deer, a species similar to juvenile red deer in size, show median blood loss of 2% of the hosts total amount of blood measured on the amount of blood in attached ticks. Extremes were reaching up to 9% blood loss. Moose (*Alces alces*), which are much larger than red deer, had only minute blood loss to ticks of about 0.1–0.3% of total blood volume.

Indirect costs are more difficult to measure. Indirect effects could among others be immunosuppression caused by tick saliva [54]–[56], reduced effort spent in feeding or other costs of avoidance [12], or tick-borne diseases like anaplasmosis [56]–[58]. *Anaplasma* sp. is known to affect livestock negatively. Anaplasmosis is known both to reduce growth and increase mortality in sheep (*Ovis aries*) in Norway [59], [60]. Though a case of a paretic condition in a young roe deer was attributed to infection with *Anaplasma* sp. [61], it is generally held that the effect of *Anaplasma* sp. on red deer is weak (S. Stuen, pers. comm.).

Abundant ectoparasites tend to alter behavior in wild animals. Little [62] found that an average infestation of 50 cattle ticks (*B. Microplus*) gave a growth reduction of 0.76 kg per engorged female tick, suggested to be mostly through indirect effects. Scratching and grooming among cervids are direct responses to infestations by ticks and other parasites [12], [55], [63]. Tick infestation could, based on clinical experience in domestic animals and humans, be expected to cause varying degree of local pruritus (“itching”) and/or pain, but to our knowledge no investigation on clinical signs of tick infestation in cervids have been reported. Pruritus and/or pain could in turn cause restlessness and reduced foraging. Ticks are likely annoying to deer, but whether or how much fitness is affected remains an open question. Both direct and indirect effects of parasites may be more severe in growing newborn and young than for the adults both in birds and mammals [54]. Norwegian red deer give birth in their summer range between 6 June and 4 July [64] after migration in May [8], hence reduced parasitism of offspring may be the more important effect. Clearly, an experimental approach measuring growth of young deer, i.e., using acaricide treatment and untreated controls, would be preferred before firm conclusion on the role of ticks for early growth can be assessed with certainty.

### Conclusion

Tick abundance is not the primary driver behind red deer migration, but our study suggests it might add to the benefit of migration in our system as migration also leads to a spatial separation from ticks. More detailed data are needed to verify whether red deer time migration to avoid main tick questing periods, or whether resident red deer avoid tick hot spots locally. Our study also demonstrates a potential function of migratory red deer to move ticks around in the landscape over long distances each spring.

## Supporting Information

Table S1 **Results from model selection performed on tick abundance from the Sogn & Fjordane and Møre & Romsdal counties in Norway at the scale of red deer home ranges, on both May and August data, and performed on different stages.**
(DOCX)

Table S2 **Parameter estimates and test statistics for all best models of tick abundance relative to red deer home ranges.** Note that all models predict fewer ticks in the summer home ranges as compared to winter home ranges and home ranges of resident red deer.
(DOCX)
