# Supplementary material for: Landscape Level Variation in Tick Abundance Relative to Seasonal Migration in Red Deer
Source: PLoS One. 2013 Aug 9;8(8):e71299. doi: 10.1371/journal.pone.0071299 (PMC3739797; doi:10.1371/journal.pone.0071299)
Supplement: Table S1 — Results from model selection performed on tick abundance from the Sogn & Fjordane and Møre & Romsdal counties in Norway at the scale of red deer home ranges, on both May and August data, and performed on different stages. (DOCX) [file pone.0071299.s001.docx]

**Table S1.** Results from model selection performed on tick abundance from the Sogn & Fjordane and Møre & Romsdal counties in Norway at the scale of red deer home ranges, on both May and August data, and performed on different stages. HRtype = home range type (3 levels), year (4 or 2 levels). The models also included red deer ID as a random term. The best models are highlighted in bold.

| HRtype | Year | Sex | HRtype :Year | HRtype:Sex | Sex:Year | | Hrtype:Sex:Year | AIC | ΔAIC |
| --- | --- | --- | --- | --- | --- | --- | --- | --- | --- |
| Sogn & Fjordane, *May, all ticks pooled* | | | | | |  | |  | |
|  |  |  |  |  |  | |  | 14455.0 | 285.3 |
| x |  |  |  |  |  | |  | 14217.4 | 47.64 |
|  | x |  |  |  |  | |  | 14457.7 | 287.94 |
| x | x |  |  |  |  | |  | 14213.6 | 43.92 |
| **x** | **x** |  | **x** |  |  | |  | **14169.7** | **0** |
| Sogn & Fjordane, *May- Adult female* | | | | | | | | | |
|  |  |  |  |  |  | |  | 2879.1 | 75.5 |
| x |  |  |  |  |  | |  | 2843.9 | 40.4 |
|  | x |  |  |  |  | |  | 2860.0 | 56.5 |
| x | x |  |  |  |  | |  | 2823.2 | 19.66 |
| **x** | **x** |  | **x** |  |  | |  | **2803.5** | **0** |
| Sogn & Fjordane, *May- Adult male* | | | | | | | | | |
|  |  |  |  |  |  | |  | 3217.1 | 74.8 |
| x |  |  |  |  |  | |  | 3183.1 | 40.9 |
|  | x |  |  |  |  | |  | 3173.9 | 31.6 |
| x | x |  |  |  |  | |  | 3147.3 | 5.1 |
| x | x |  | x |  |  | |  | **3142.2** | **0** |
| Sogn & Fjordane, *August* | | | | | | | | | |
|  |  |  |  |  |  | |  | 8908.0 | 75.8 |
| x |  |  |  |  |  | |  | 8855.1 | 22.9 |
|  | x |  |  |  |  | |  | 8886.2 | 54 |
| **x** | **x** |  |  |  |  | |  | **8833.3** | **1.1** |
| x | x |  | x |  |  | |  | 8832.2 | 0 |
| Sogn & Fjordane, *August****-*** *Adult female* | | | | | | | | | |
|  |  |  |  |  |  | |  | 2163.3 | 35.0 |
| x |  |  |  |  |  | |  | 2149.7 | 21.7 |
|  | x |  |  |  |  | |  | 2151.3 | 23.3 |
| x | x |  |  |  |  | |  | 2137.8 | 9.8 |
| **x** | **x** |  | **x** |  |  | |  | **2128.0** | **0** |
| Sogn & Fjordane, *August- Adult male* | | | | | | | | | |
|  |  |  |  |  |  | |  | 2217.7 | 18.5 |
| x |  |  |  |  |  | |  | 2208.5 | 9.2 |
|  | x |  |  |  |  | |  | 2211.8 | 12.6 |
| x | x |  |  |  |  | |  | 2202.5 | 3.2 |
| **x** | **x** |  | **x** |  |  | |  | **2199.3** | **0** |
| Møre & Romsdal, *May* | | | | | | | | | |
|  |  |  |  |  |  | |  | 5900.9 | 45.3 |
| x |  |  |  |  |  | |  | 5869.9 | 14.3 |
|  | x |  |  |  |  | |  | 5897.2 | 41.6 |
|  |  | x |  |  |  | |  | 5902.5 | 46.9 |
| x | x |  |  |  |  | |  | 5870.4 | 14.8 |
| x |  | x |  |  |  | |  | 5873.5 | 17.9 |
|  | x | x |  |  |  | |  | 5898.7 | 43.1 |
| x | x | x |  |  |  | |  | 5872.4 | 16.8 |
| **x** | **x** |  | **x** |  |  | |  | **5855.6** | **0** |
| x |  | x |  | x |  | |  | 5874.0 | 18.4 |
|  | x | x |  |  | x | |  | 5894.5 | 38.9 |
| x | x | x | x |  |  | |  | 5857.6 | 2.0 |
| x | x | x |  | x |  | |  | 5874.3 | 18.7 |
| x | x | x |  |  | x | |  | 5868.3 | 12.7 |
| x | x | x | x | x |  | |  | 5859.6 | 4.0 |
| x | x | x | x |  | x | |  | 5855.8 | 0.2 |
| x | x | x |  | x | x | |  | 5870.2 | 14.6 |
| x | x | x | x | x | x | |  | 5857.8 | 2.2 |
| x | x | x | x | x | x | | x | 5861.4 | 5.8 |
| Møre & Romsdal, *August* | | | | | | | | | |
|  |  |  |  |  |  | |  | 5533.9 | 271.9 |
| x |  |  |  |  |  | |  | 5271.4 | 9.4 |
|  | x |  |  |  |  | |  | 5527.1 | 265.1 |
| x | x |  |  |  |  | |  | 5270.2 | 8.2 |
| **x** | **x** |  | **x** |  |  | |  | **5262.0** | **0** |
| Møre & Romsdal, *August, adults* | | | | | | | | | |
|  |  |  |  |  |  | |  | 1618.9 | 64.1 |
| **x** |  |  |  |  |  | |  | **1554.8** | 0 |
|  | x |  |  |  |  | |  | 1620.8 | 66.0 |
| x | x |  |  |  |  | |  | 1556.7 | 1.9 |
| x | x |  | x |  |  | |  | 1558.1 | 3.3 |
