# Supplementary material for: Landscape Level Variation in Tick Abundance Relative to Seasonal Migration in Red Deer
Source: PLoS One. 2013 Aug 9;8(8):e71299. doi: 10.1371/journal.pone.0071299 (PMC3739797; doi:10.1371/journal.pone.0071299)
Supplement: Table S2 — Parameter estimates and test statistics for all best models of tick abundance relative to red deer home ranges. Note that all models predict fewer ticks in the summer home ranges as compared to winter home ranges and home ranges of resident red deer. (DOCX) [file pone.0071299.s002.docx]

**Table S2.** Parameter estimates and test statistics for all best models of tick abundance relative to red deer home ranges. Baseline year for Sogn & Fjordane is 2009, while it is 2011 for Møre og Romsdal county. Note that all models predict fewer ticks in the summer home ranges as compared to winter home ranges and home ranges of resident red deer, though not quite significant for the Sogn & Fjordane August adult male model due to low sample size. Models using only adult stages in Møre & Romsdal as response had problems converging due to a small amount of data. Adult males and females are therefore pooled in this area. Note that the models where all tick stages are pooled show the results from the bootstrap analysis.

| **Sogn & Fjordane***, May, all ticks pooled, and bootstrapped* | Estimate | S.E. | z | p |
| --- | --- | --- | --- | --- |
| Intercept | -8e-3 | 0.33 | -0.0 | 0.982 |
| HR (resident vs. summer) | 1.3 | 0.39 | 3.5 | 0.008 |
| HR (winter vs. summer) | 1.3 | 0.17 | 7.7 | <0.001 |
| Year 2010 vs. 2009 | -0.3 | 0.15 | -2.1 | 0.035 |
| Year 2011 vs. 2009 | -0.6 | 0.16 | -3.6 | 0.002 |
| Year 2012 vs. 2009 | 0.08 | 0.16 | -0.5 | 0.632 |
| HR (resident vs. summer): Year 2010 vs. 2009 | 0.3 | 0.19 | 1.6 | 0.106 |
| HR (resident vs. summer): Year 2011 vs. 2009 | 6e-3 | 0.20 | -0.0 | 0.973 |
| HR (resident vs. summer): Year 2012 vs. 2009 | -0.2 | 0.20 | -1.0 | 0.305 |
| HR (winter vs. summer):Year 2010 vs. 2009 | 0.5 | 0.21 | 2.3 | 0.021 |
| HR (winter vs. summer):Year 2011 vs. 2009 | 1.1 | 0.22 | 4.9 | <0.001 |
| HR (winter vs. summer):Year 2012 vs. 2009 | 0.02 | 0.21 | -0.1 | 0.905 |
| **Sogn & Fjordane***, May- Adult female* | Estimate | S.E. | z | p |
| Intercept | -3.0 | 0.36 | -8.5 | <0.001 |
| HR (resident vs. summer) | 1.2 | 0.49 | 2.4 | 0.015 |
| HR (winter vs. summer) | 1.1 | 0.17 | 6.1 | <0.001 |
| Year 2011 | -0.7 | 0.15 | -4.3 | <0.001 |
| Year 2010 | -0.09 | 0.13 | -0.7 | 0.518 |
| Year 2012 | -0.5 | 0.15 | -3.5 | <0.001 |
| **Sogn & Fjordane***, May- Adult male* | Estimate | S.E. | z | p |
| Intercept | -2.3 | 0.50 | -4.6 | <0.001 |
| HR (resident vs. summer) | 1.3 | 0.39 | 3.2 | 0.001 |
| HR (winter vs. summer) | 0.9 | 0.18 | 5.1 | <0.001 |
| Year 2010 | -0.2 | 0.14 | -1.5 | 0.123 |
| Year 2011 | -1.0 | 0.16 | -6.4 | <0.001 |
| Year 2012 | -0.6 | 0.14 | -4.5 | <0.001 |
| **Sogn & Fjordane***, August, all ticks pooled and bootstrapped* | Estimate | S.E. | z | p |
| Intercept | -0.2 | 0.28 | -0.9 | 0.375 |
| HR (resident vs. summer) | 1.1 | 0.36 | 3.1 | 0.002 |
| HR (winter vs. summer) | 0.70 | 0.092 | 7.5 | <0.001 |
| Year 2011 | -0.35 | 0.074 | -4.8 | <0.001 |
| Year 2012 | -0.42 | 0.073 | -5.8 | <0.001 |
| **Sogn & Fjordane***, August- Adult female* | Estimate | S.E. | z | p |
| Intercept | -3.1 | 0.37 | -8.3 | <0.001 |
| HR (resident vs. summer) | 1.4 | 0.50 | 2.9 | 0.004 |
| HR (winter vs. summer) | 0.9 | 0.29 | 3.1 | 0.002 |
| Year 2011 | -1.5 | 0.47 | -3.3 | 0.001 |
| Year 2012 | 0.4 | 0.29 | 1.2 | 0.222 |
| Resident : 2011 | 1.2 | 0.52 | 2.4 | 0.018 |
| Winter : 2011 | 0.8 | 0.55 | 1.5 | 0.145 |
| Resident : 2012 | -0.6 | 0.35 | -1.7 | 0.099 |
| Winter : 2012 | -0.9 | 0.40 | -2.2 | 0.026 |
| **Sogn & Fjordane***, August- Adult male* | Estimate | S.E. | z | p |
| Intercept | -2.7 | 0.33 | -8.1 | <0.001 |
| HR (resident vs. summer) | 0.7 | 0.45 | 1.6 | 0.118 |
| HR (winter vs. summer) | -0.02 | 0.30 | -0.1 | 0.930 |
| Year 2011 | -1.1 | 0.37 | -3.1 | 0.002 |
| Year 2012 | -0.3 | 0.29 | -1.0 | 0.304 |
| Resident : 2011 | 1.3 | 0.42 | 3.0 | 0.003 |
| Winter : 2011 | 1.2 | 0.47 | 2.4 | 0.015 |
| Resident : 2012 | 0.8 | 0.35 | 2.3 | 0.023 |
| Winter : 2012 | 0.7 | 0.40 | 1.8 | 0.071 |
| **Møre & Romsdal***, May, all ticks pooled and bootstrapped* | Estimate | S.E. | z | p |
| Intercept | -0.6 | 0.33 | -1.7 | 0.085 |
| HR (resident vs. summer) | 2.7 | 0.54 | 4.8 | <0.001 |
| HR (winter vs. summer) | 2.6 | 0.20 | 12 | <0.001 |
| Year 2012 vs. 2011 | 0.5 | 0.17 | 3.1 | 0.002 |
| HR (resident vs. summer):Year | -0.6 | 0.25 | -2.2 | 0.036 |
| HR (winter vs. summer):Year | -0.9 | 0.24 | -3.9 | <0.001 |
| **Møre & Romsdal**, *May, adults* | Estimate | S.E. | z | p |
| Intercept | -2.9 | 0.43 | -6.6 | <0.001 |
| HR (resident vs. summer) | 2.4 | 0.45 | 5.3 | <0.001 |
| HR (winter vs. summer) | 2.8 | 0.46 | 6.2 | <0.001 |
| Year 2012 vs. 2011 | 1.3 | 0.37 | 3.5 | <0.001 |
| HR (resident vs. summer):Year | -1.0 | 0.44 | -2.2 | 0.029 |
| HR (winter vs. summer):Year | -2.0 | 0.49 | -4.0 | <0.001 |
| **Møre & Romsdal***, August, all ticks pooled and bootstrapped* | Estimate | S.E. | z | p |
| Intercept | -0.6 | 0.32 | -1.8 | 0.090 |
| HR (resident vs. summer) | 2.0 | 0.53 | 3.7 | <0.001 |
| HR (winter vs. summer) | 2.0 | 0.19 | 10.3 | < 2e-16 |
| Year 2012 vs. 2011 | 0.2 | 0.17 | 1.2 | 0.304 |
| HR (resident vs. summer):Year | -0.15 | 0.24 | -0.6 | 0.469 |
| HR (winter vs. summer):Year | -0.35 | 0.23 | -1.55 | <0.001 |
| ***Møre & Romsdal****, August, adults* | Estimate | S.E. | z | p |
| Intercept | -2.5 | 0.24 | -10.62 | <0.001 |
| HR (resident vs. summer) | 1.5 | 0.33 | 4.4 | <0.001 |
| HR (winter vs. summer) | 1.4 | 0.18 | 7.54 | <0.001 |
